# Longitudinal Choriocapillaris Vascular Density Changes in Different Types of Primary Open-Angle Glaucoma

**DOI:** 10.1167/tvst.12.1.21

**Published:** 2023-01-18

**Authors:** Weijing Cheng, Yunhe Song, Fei Li, Fengbin Lin, Bin Yang, Fanyin Wang, Guili Ning, Hao Li, Wei Wang, Xiulan Zhang

**Affiliations:** 1 State key Laboratory of Ophthalmology, Zhongshan Ophthalmic Center, Sun Yat-sen University, Guangzhou, China; 2 Department of Ophthalmology, Zigong Third People's Hospital, Zigong, China; 3 Department of Ophthalmology, Shenzhen Qianhai Shekou Free Trade Zone Hospital, Shenzhen, China; 4 Department of Ophthalmology, Guizhou Aerospace Hospital, Zunyi, China; 5 Department of Ophthalmology, Guizhou Provincial People's Hospital, Guiyang, China

**Keywords:** optical coherence tomography angiography, glaucoma, choriocapillaris vascular density, optic disc phenotypes

## Abstract

**Purpose:**

To evaluate longitudinal changes in choriocapillaris perfusion in patients with glaucoma with four phenotypes of optic disc damage and to explore associated factors with decreased choriocapillaris vessel density (CVD).

**Methods:**

This prospective longitudinal study included 96 eyes of 96 patients with primary open-angle glaucoma (POAG). Patients with POAG was differentiated into the optic disc phenotypes of focal ischemic type (FI), myopic type (MY), senile sclerotic type (SS), and generalized enlargement type (GE). Patients were followed up every three months. Simple linear regression was used to investigate the factors associated with a reduction in CVD.

**Results:**

The median follow-up time was 2.5 years (range, 2.0–3.0 years). Choriocapillaris perfusion tended to decrease over time, with CVD decreasing significantly faster in the FI type than in the other three types (*P* < 0.001). The percentage decrease in the FI type was 7.85%, 10.89%, and 8.88% faster than MY, SS and GE, respectively, after correcting for age, gender, axial length, intraocular pressure, mean deviation, retinal nerve fiber layer (RNFL), and image quality score. In multivariate regression, decreased CVD was independently associated with the rate of RNFL thinning.

**Conclusions:**

FI type had the fastest rate of CVD decline in the four phenotypes of optic disc damage, and decreased CVD was positively correlated with the rate of RNFL thinning.

**Translational Relevance:**

The role of the choriocapillaris in the pathogenesis and therapeutic potential of glaucoma require further attention to facilitate better management of glaucoma patients.

## Introduction

Glaucoma affects about 64 million people worldwide,^1^ and it is characterized by retinal ganglion cell damage and optic nerve degeneration. The glaucomatous optic nerve damage can be differentiated into four phenotypes: focal ischemic type (FI), myopic type (MY), senile sclerotic type (SS), and generalized enlargement type (GE).^2^^–^^11^ These types differ in their clinical features, including retinal nerve fiber layer (RNFL) thickness and ganglion cell–inner plexiform layer (GCIPL) thickness, therapeutic responses, and progression of visual field loss.^2^^–^^9^ Nicolela and colleagues^3^ reported that the FI type showed the fastest visual field deterioration, with a 37%, 86%, and 6% higher risk of progression as compared to the MY type, SS type, and GE type. Spaeth and coworkers^12^ found that the FI type responded better to a reduction in intraocular pressure (IOP) than the other types. The findings were the basis for the notion that the pathogenesis of optic nerve damage differs between the different glaucoma types.

With the mechanism of glaucomatous nerve damage not yet well understood, ischemia has been considered to play a role in the pathogenesis of glaucomatous optic neuropathy (GON).^13^ The choroid receives 80% to 90% of the ocular blood flow and nourishes the outer retinal layer.^14^^,^^15^ Until recently, the choriocapillaris perfusion, however, was not yet quantified in vivo at high resolution because of limitations of the imaging techniques.^16^ The conventional indocyanine green angiography (ICGA) was the gold standard for the examination of the choroidal blood system but had limitations in visualizing the choriocapillaris.^15^^,^^17^^–^^19^ In addition, ICGA is an invasive technique. These were the reasons why the choriocapillaris has not been in the different phenotypes of glaucoma.

The clinical introduction of swept source optical coherence tomography angiography (SS-OCTA) technology has made it possible to visualize noninvasively the choriocapillaris in vivo and allows quantitative measurements. Standard choriocapillaris analysis methods based on SS-OCTA have been established and successfully applied in diabetic retinopathy, central serous chorioretinopathy, and age-related macular degeneration.^20^^–^^23^ Studies on differences in choriocapillaris and choriocapillaris changes in follow-up examinations have not been performed for the four glaucomatous phenotypes. This study therefore analyzed longitudinal changes of choriocapillaris in the four glaucoma phenotypes.

## Methods

This prospective longitudinal study was conducted at the Clinical Research Center of Zhongshan Ophthalmic Center, Sun Yat-sen University. The study protocol was approved by the ethics committee of Zhongshan Ophthalmic Center. The study followed the tenets of the Declaration of Helsinki. Written informed consent was obtained from all subjects before enrollment.

### Study Population

Patients with clinically confirmed primary open angle glaucoma (POAG) were included in this study and met the following criteria: (1) untreated IOP of ≥21 mm Hg with typical glaucomatous visual field defects and GON. Visual field defects were defined as a glaucomatous hemifield VF defect in the superior or inferior hemifield according to the Anderson-Patellar criteria,^24^ which was confirmed by at least two reliable visual field examinations. GON was defined as a vertical cup-to-disc ratio ≥0.7 (unless explained by a large optic disc), or a cup-to-disc ratio asymmetry >0.2, neuroretinal rim thinning, and RNFL defect; (2) open angle confirmed by gonioscopy; (3) best corrected visual acuity ≥20/30; (4) refractive error (spherical equivalent) >+2.0 diopters (D) or <−6.0 D; (5) axial length <26 mm; (6) could follow up every three months for a period of at least 2 years; and (7) IOP is stable within the range of 10–21 mm-Hg under treatment with IOP-lowering eye drops.

Exclusion criteria were: (1) history of primary angle closure glaucoma, secondary glaucoma (e.g., exfoliative or pigmentary glaucoma), or congenital glaucoma; (2) history of intraocular surgery or laser treatment; (3) other ocular diseases, such as trauma, keratitis, uveitis, cataract, age-related degeneration, diabetic retinopathy, retinal vein occlusion, the retinal anterior membrane, macular edema, etc.; (4) history of cerebrovascular events (e.g., Parkinson's disease, Alzheimer's disease, dementia or history of stroke, etc.) or history of other cardiovascular diseases, diabetes mellitus, hypertension, or with systematic measurements that may influence the final microscopic measurement, such as chloroquine/hydroxychloroquine, glucocorticoid, chlorpromazine, thiolidazine, tamoxifen, canthaxanthin, sildenafil, and niacin; and (5) inability to cooperate with all ophthalmic examinations or poor image quality. Only the right eye of the individuals was included in the study.

### Follow-Up and Baseline Examinations

All enrolled glaucoma patients underwent a comprehensive baseline examination and follow-up examinations at three-month intervals. Anterior and posterior segmental examinations were performed using a slit lamp (BQ-900; Haag-Streit AG, Koeniz, Switzerland) and an ophthalmoscope. The gonioscopy examination was conducted as a baseline before enrollment. Blood pressure was measured twice using an Omron automated sphygmomanometer. Ocular parameters, including axial length (AL), lens thickness (LT), and anterior chamber depth (ACD), were measured using optical coherence biometry (IOL master 500; Carl Zeiss Meditec, Jena, Germany). IOP was measured using a Goldmann tonometer. Visual field examination was performed using a Humphrey Field Analyzer with 24-2 Swedish Interactive Threshold Algorithm standard strategies (Carl Zeiss Meditec). The first visual field examination for each patient was excluded because of the potential learning effects of the visual field examinations. Visual field reports were checked for artifacts, including fatigue, inattention, poor vision, and eyelid or eyelash artifacts, and visual field results with such artifacts were excluded. Reliable visual field reports, including fixation loss rate ≤33%, false-positive error rates ≤15% and false-negative error rates ≤15%. All ocular examinations were performed in the order of the right and left eyes. All patients underwent a standard questionnaire (The China Health and Retirement Longitudinal Study) simultaneously, including a history of systemic and ocular disease, surgical history, history of medication, obstructive sleep apnea syndrome and migraine.

### Fundus Examination and Classification of The Optic Disc Phenotype

**Figure 1.**
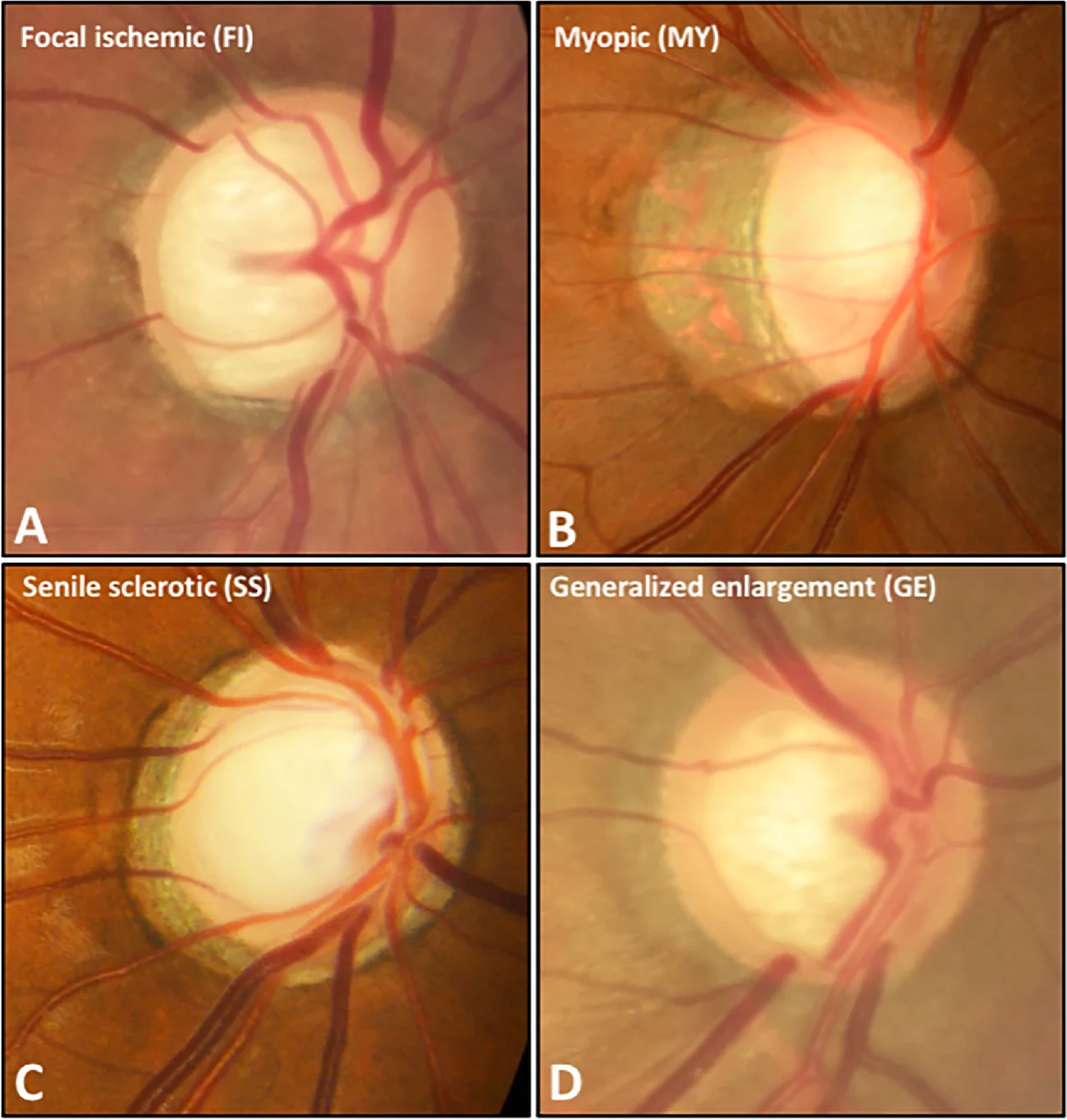
Representative optic disc photographs of the eye with four glaucomatous optic disc phenotypes. (**A**) FI type. (**B**) MY type. (**C**) SS type. (**D**) GE type.

Stereoscopic color fundus photographs were obtained after medical induction of mydriasis using a fundus stereoscopic camera (Nonmyd WX3D; Kowa, Nagoya, Japan). Four phenotypes were classified according to previous reports (Fig. 1).^2^^–^^10^ (1) There was a GE type with concentric enlargement of the cup, no limited defect of the neuroretinal rim and no obvious chorioretinal atrophy. (2) In the FI type, the optic disc showed a deep localized defect in the neuroretinal rim, whereas the rest of the neuroretinal rim was not markedly diminished, without a significant chorioretinal atrophy. (3) In the MY type, usually seen in myopic patients with mild tilting of the optic disc with temporal crescentic atrophy and thinning of the retinal nerve margins temporally, inferiorly and/or superiorly. (4) Patients with the SS type had a circular parapapillary beta zone, with shallow cupping and a “moth-eaten” pale appearance of the neuroretinal rim. (5) In the case of a combined occurrence of various optic disc phenotypes, the optic disc damage was classified as a mixed-type. (6) The remaining optic discs that were considered to have a normal appearance or small optic discs or could not be classified were classified as “others.” Two glaucoma specialists, masked to the participants' identity, diagnostic status, and other information, independently of each other classified the optic disc damage type based on the optic disc photographs. If they did not agree in the classification, the examinations were repeated under the guidance of a professor of glaucoma (Z.X.L.), and a consensus was finally reached.

### SS-OCTA Imaging and Analysis

**Figure 2.**
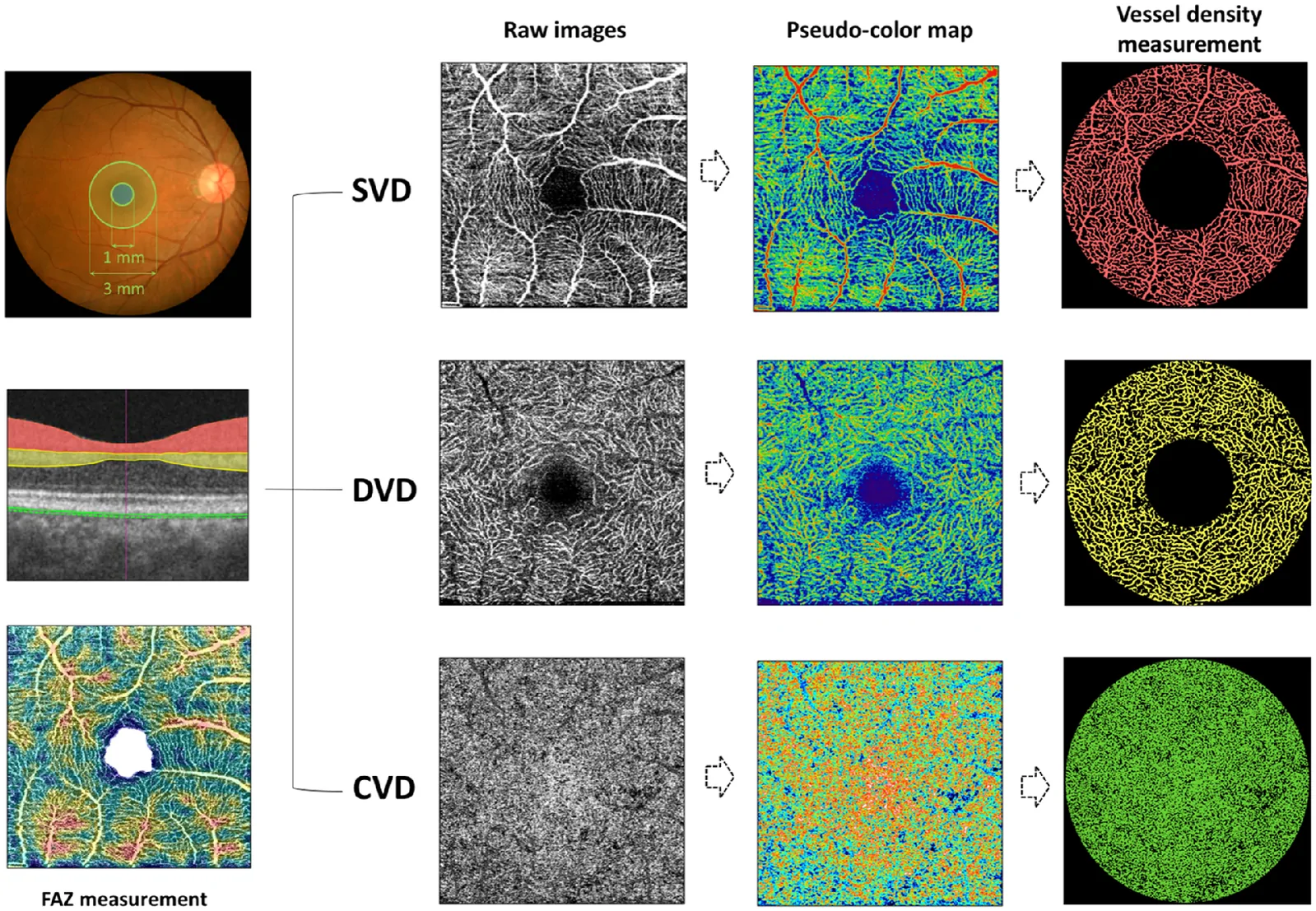
Measurement of foveal avascular zone, superficial vessel density, deep vessel density and choriocapillaris vessel density. FAZ, foveal avascular zone.

The SS-OCT (DRI-OCT-2; Topcon, Tokyo, Japan) was used to obtain high-resolution images of blood vessels, peripapillary RNFL thickness, and mean choroidal thickness. The instrument used laser beam with a wavelength of 1050 nm, a scanning speed of 100,000 A-scans/s, and an axial resolution of 8 µm. The 3D volume scan was performed using a 3 × 3 mm^2^ raster scanning protocol (256 × 256 pixels) centered at the fovea. During the imaging, an eye-tracking system was used to reduce motion artifacts. Projection artifact removal was also available in the software and was activated. The macula was divided into four layers by automatic segmentation software: superficial capillary plexus (SCP), deep capillary plexus (DCP), outer retina, and choriocapillaris (CC). Automatic segmentation was performed using IMAGEnet 6 (version 1.23; Topcon), defining the boundaries of the SCP from 2.6 µm below the internal limiting membrane to 15.6 µm below the inner plexiform layer and inner nuclear layer (IPL/INL); the DCP from 15.6 µm below the IPL/INL to 70.2 µm below the IPL/INL; the CC from the Bruch membrane (BM; 0 mm offset at BM setting) to 10.4 µm below the BM. The images were obtained in a dark room by three experienced technicians. If multiple OCTA images were obtained for one eye, then the one with the highest image quality score was retained. ImageNet (Topcon) was used to output images of each layer for further manual analysis of vascular parameters, and Image J (National Institutes of Health, Bethesda, MD, USA) was applied according to the standard methods previously reported^25^^,^^26^ (Fig. 2). The images were adjusted for magnification effects using Littmann's and Bennett's formula^25^^,^^27^^,^^28^ and then automatically binarized after feature extraction to remove white abnormal noise. Finally, white pixels as a percentage of the total area were automatically measured as the superficial vessel density (SVD) and deep vessel density (DVD) layers. After binarization (“Huang” method), the outline of the foveal avascular zone was manually drawn using Image J. As reported previously, CC images were measured according to the Guidelines for Imaging the Choriocapillaris Using OCT Angiography.^23^ Images were binarized using the Phansalkar method (a window radius of six pixels) to quantify the proportion of white pixels in the measurement area, which was defined as the choriocapillaris vessel density (CVD). Two senior investigators performed the quality control of all OCTA scans based on subject characteristics. Low-quality OCTA images that met one of the following criteria were excluded: (1) image quality score less than 40; (2) motion artifacts (e.g., discontinuous vessels or distinct white lines of motion); (3) segmentation errors; (4) blurred images (e.g., only large vessels are visible and capillaries are not clearly visible); (5) masking artifacts (e.g., caused by refractive media opacity); and (6) decentration artifacts (i.e., the fovea was not centered at the image).

### Statistical Analysis

For demographic and ocular characteristics, categorical variables were compared using the χ^2^ test, normally distributed continuous variables were compared using the two-tailed *t*-test, and non-normally distributed variables were compared using the Wilcoxon rank sum test. Simple linear regression was used to calculate the absolute change and percentage change of OCTA parameters (with baseline measurements as the denominator). Bonferroni correction was considered to adjust for multiple comparisons in statistical significance (*P* = 0.05/6 = 0.008 with Bonferroni adjustment). Univariate and multivariate linear models were used to investigate the factors affecting CVD reduction. Parameters with a *P* value <0.1 in the univariate analysis were included in the multivariate regression analysis. Intraclass correlation coefficient (ICC) were used to assess agreement of optic disc phenotype. ICC values of less than 0.5, between 0.5 and 0.75, between 0.75 and 0.9, and greater than 0.90 indicated poor, moderate, good, and excellent repeatability, respectively. All statistical analyses were performed using STATA software version 16.0 (Stata Corporation, College Station, TX, USA).

## Results

Table 1 shows the baseline demographic and ocular characteristics of the study population. Mixed-type and others (n = 76) were excluded (see flow chart in Supplementary Fig. S1). In the final analysis, a total of 96 eligible patients were included, with a mean age of 51.3 ± 10.2, and 22 (22.7%) were female. The median follow-up time was 2.5 years (range, 2–3 years). During the two- to three-year follow-up period, the patients were followed-up every three months. The average number of images of each patient was 11; of these, 7.16 ± 1.13 met the quality control criteria (Supplementary Table S1). The SS type glaucoma subjects were older, the MY type had a longer AL, the GE type had the thickest RNFL and the FI type had the thickest choroidal thickness (all *P* < 0.05). There were no significant differences in gender, systolic blood pressure, diastolic blood pressure, LT, ACD, central corneal thickness, mean IOP during follow-up, mean deviation (MD) of the visual field, a standard deviation of visual field pattern and image quality score among participants with the four phenotypes of glaucoma. The initial IOP of type FI was 22.81 ± 1.56 mm Hg, that of type MY was 23.38 ± 1.45 mm Hg, that of type SS was 25.52 ± 2.92 mm Hg, and that of type GE was 24.31 ± 1.01 mm Hg. There were one, two, and one obstructive sleep apnea syndrome patients in the FI, SS, and GE groups, respectively. And there were two, one, three, and one migraine patients in the FI, MY, SS, and GE groups, respectively.

**Table 1.** Baseline Demographics and Ocular Characteristics of Study Population

Characteristic	FI	MY	SS	GE	*P* Value^*^
No. of subjects (eyes)	33 (33)	35 (35)	14 (14)	14 (14)	—
Mean age (y)	46.94 (6.52)	47.94 (5.29)	59.36 (13.58)	52.43 (11.29)	**0.038**
Female, no. (%)	7 (21.21%)	11 (31.43%)	1 (7.14%)	3 (21.43%)	0.323
Systolic blood pressure (mm Hg)	122.30 (11.48)	115.73 (10.85)	116.67 (10.12)	121.00 (25.16)	0.680
Diastolic blood pressure (mm Hg)	70.20 (10.48)	67.55 (7.39)	63.67 (4.93)	76.67 (21.39)	0.448
Anterior central chamber (mm)	3.49 (0.44)	3.62 (0.46)	3.26 (0.38)	3.37 (0.45)	0.056
Lens thickness (mm)	4.35 (0.59)	4.15 (0.58)	4.45 (0.91)	4.19 (0.78)	0.891
Axial length (mm)	25.12 (1.75)	26.16 (1.63)	25.08 (1.51)	24.12 (1.85)	**0.002**
Central corneal thickness (µm)	534.86 (20.23)	547.98 (38.36)	545.27 (50.54)	546.23 (28.93)	0.866
Intraocular pressure^†^ (mm Hg)	16.42 (4.24)	13.97 (2.64)	17.48 (3.10)	14.98 (4.65)	0.099
Mean deviation (dB)	−4.31 (3.68)	−6.52 (7.56)	−9.69 (11.05)	−5.93 (8.72)	0.276
Pattern standard deviation (dB)	4.33 (3.59)	4.66 (3.20)	5.45 (4.2)	4.00 (3.97)	0.811
Average pRNFL thickness (µm)	83.17 (19.88)	71.35 (20.09)	79.23 (15.75)	95.75 (12.29)	**0.001**
Average mCT (µm)	248.46 (82.74)	195.52 (74.79)	207.86 (94.99)	204.29 (64.54)	**0.049**
Image quality score of OCTA	55.61 (3.43)	59.31 (6.49)	56.21 (5.34)	57.43 (4.93)	0.171

pRNFL, peripapillary retinal nerve fiber layer thickness; mCT, macular choroidal thickness.

Data are expressed as the mean (SD) or mean (%).

* Bold indicates statistical significance.

† All eyes had been receiving multiple IOP-lowering eye drops.

The baseline vascular parameters of the four types are shown in Supplementary Table S2. Compared to the other types, the baseline foveal avascular zone area of the MY type was the largest (*P* = 0.013), the baseline of CVD was the lowest in the SS type (*P* = 0.003), and there was no significant difference in SVD and DVD between the four optic disc phenotypes. ICC on the assessment of optic disc phenotype was very good (0.86 [confidence interval, 0.72 to 0.89]) (data not shown). The number of OCTA visits and the follow-up duration of different optic disc phenotypes are provided in Supplementary Table S3. No significant differences were found between the number of OCTA visits (*P* = 0.993) and follow-up duration (*P* = 0.992) among different optic disc phenotypes.

**Figure 3.**
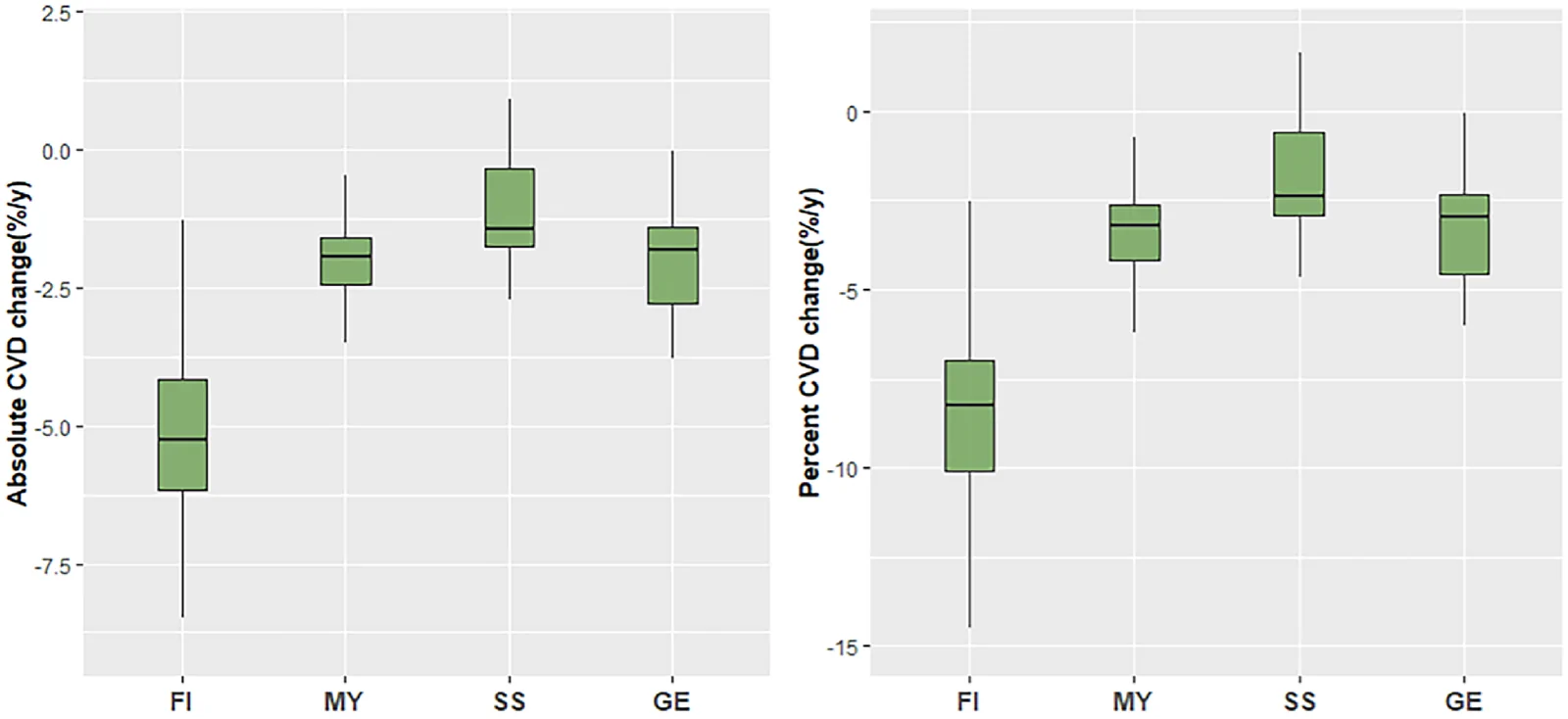
Absolute and percent changes of choriocapillaris vessel density in four glaucomatous optic disc phenotypes.

Table 2 shows the absolute and percent rates of decline in vascular parameters in each layer of the four phenotypes of glaucoma. Ocular perfusion parameters tended to decrease over time, with the FI type having a significantly faster rate of decline in CVD than in the other three types. The absolute rates of decline for FI, MY, SS and GE was −6.07%/y (−7.18%/y, −4.95%/y), −1.4%/y (−2.13%/y, −0.66%/y), 0.44%/y (−1.90%/y, 2.79%/y), and −1.01%/y (−2.48, 0.46), respectively (*P* < 0.001, with Bonferroni correction). Additionally, the percent rates of decline for FI, MY, SS and GE were 10.01%/y (−11.87%/y, −8.15%/y), −2.27%/y (−3.54%/y, −1.01%/y), 1.21%/y (−3.42%/y, 5.83%/y), and −1.55%/y (−4.08%/y, 0.98%/y) (*P* < 0.001, with Bonferroni correction), respectively. Figure 3 shows the absolute and percent rates of decline in CVD per year for each optic disc phenotype, with the FI phenotype showing the fastest decline in CVD during the two-year follow-up period.

**Table 2.** Absolute and Percent Changes of Retinal and Choroid Vascular Parameter During Follow-up in Four Glaucomatous Optic Disc Phenotypes

	FI	MY	SS	GE	*P* Value^*^
Absolute rate					
FAZ (mm^2^/y)	0.08 (0.002, 0.16)	0.02 (−0.02, 0.07)	0.04 (0.001, 0.08)	0.001 (−0.05, 0.05)	0.27
SVD (%/y)	−1.31 (−3.73, 1.10)	−2.03 (−3.35, −0.71)	−0.95 (−3.24, 1.34)	−0.23 (−3.70, 3.25)	0.75
DVD (%/y)	−3.38 (−6.16, −0.6)	−2.40 (−3.58, −1.23)	0.26 (−3.29, 3.80)	−4.12 (−8.81, 0.57)	0.25
CVD (%/y)	−6.07 (−7.18, −4.95)	−1.40 (−2.13, −0.66)	0.44 (−1.90, 2.79)	−1.01 (−2.48, 0.46)	**<0.001**
Percent rate					
FAZ (%/y)	41.27 (−3.73, 86.27)	11.69 (−6.28, 29.66)	12.92 (0.70, 25.15)	−2.59 (−20.6, 15.43)	0.27
SVD (%/y)	−3.10 (−9.06, 2.87)	−4.91 (−8.17, −1.64)	−2.18 (−7.92, 3.57)	−0.44 (−9.03, 8.15)	0.74
DVD (%/y)	−8.65 (−16.07, −1.23)	−5.99 (−8.97, −3.02)	1.41 (−8.94, 11.76)	−10.37 (−21.73, 0.99)	0.22
CVD (%/y)	−10.01 (−11.87, −8.15)	−2.27 (−3.54, −1.01)	1.21 (−3.42, 5.83)	−1.55 (−4.08, 0.98)	**<0.001**

FAZ, foveal avascular zone.

Data are expressed as the estimates (95% CI).

* Bold indicates statistical significance.

To exclude the potential effects of confounding factors, two multivariate models were further performed. Both models (Model 1 was corrected for age, and gender; Model 2 was further adjusted for AL, IOP, MD, RNFL and image quality score) showed that CVD in the FI type declined more rapidly than MY, SS and GE type (Table 3) by 7.51%, 12.01%, and 8.62% (Model 1) and 7.85%, 10.89%, and 8.88% (Model 2). We found that CVD in the MY type declined more rapidly than that in the SS type by 2.42% (absolute rate) and 4.50% (percent rate) in Model 1, and there was no significant difference in the rate of change in Model 2 (Supplementary Table S4).

**Table 3.** Changes of Vascular Parameters in Four Glaucomatous Optic Disc Phenotypes After Adjusted for Potential Confounder

	Model 1^*^			Model 2^†^		
	MY vs FI	SS vs FI	GE vs FI	MY vs FI	SS vs FI	GE vs FI
Absolute rate						
FAZ (mm^2^/y)	−0.05 (−0.13, 0.03) *P* = 0.190	−0.01 (−0.12, 0.09) *P* = 0.822	−0.07 (−0.16, 0.03) *P* = 0.183	−0.01 (−0.11, 0.09) *P* = 0.815	0.07 (−0.13, 0.26) *P* = 0.477	−0.03 (−0.14, 0.09) *P* = 0.620
SVD (%/y)	−1.00 (−3.53, 1.53) *P* = 0.435	−0.54 (−3.98, 2.90) *P* = 0.757	0.76 (−2.61, 4.13) *P* = 0.654	0.53 (−3.40, 4.46) *P* = 0.787	2.62 (−4.00, 9.23) *P* = 0.429	−0.98 (−6.05, 4.09) *P* = 0.698
DVD (%/y)	1.11 (−1.98, 4.20) *P* = 0.477	3.00 (−1.23, 7.24) *P* = 0.162	−0.89 (−4.95, 3.17) *P* = 0.664	0.58 (−4.46, 5.61) *P* = 0.818	10.88 (2.27, 19.50) ***P* = 0.014**	1.26 (−5.34, 7.85) *P* = 0.702
CVD (%/y)	4.53 (3.15, 5.91) ***P* < 0.001**	6.96 (5.06, 8.85) ***P* < 0.001**	5.14 (3.33, 6.96) ***P* < 0.001**	4.76 (2.89, 6.62) ***P* < 0.001**	6.54 (3.35, 9.73) ***P* < 0.001**	5.23 (2.79, 7.67) ***P* < 0.001**
Percent rate						
FAZ (%/y)	−21.60 (−61.10, 17.90) *P* = 0.278	−10.5 (−64.50, 43.50) *P* = 0.699	−33.68 (−84.50, 17.20) *P* = 0.191	−9.35 (−47.10, 28.50) *P* = 0.619	23.45 (−52.05, 98.90) *P* = 0.531	−10.7 (−56.00, 34.60) *P* = 0.634
SVD (%/y)	−2.49 (−8.76, 3.77) *P* = 0.431	−1.30 (−9.82, 7.21) *P* = 0.762	1.86 (−6.48, 10.19) *P* = 0.659	1.35 (−8.35, 11.06) *P* = 0.780	6.77 (−9.56, 23.10) *P* = 0.407	−2.44 (−14.95, 10.06) *P* = 0.695
DVD (%/y)	3.08 (−5.06, 11.21) *P* = 0.455	8.57 (−2.60, 19.74) *P* = 0.131	−2.03 (−12.73, 8.68) *P* = 0.708	1.23 (−12.35, 14.80) *P* = 0.856	31.26 (8.03, 54.50) ***P* = 0.010**	3.12 (−14.67, 20.91) *P* = 0.725
CVD (%/y)	7.51 (5.06, 9.97) ***P* < 0.001**	12.01 (8.64, 15.38) ***P* < 0.001**	8.62 (5.39, 11.86) ***P* < 0.001**	7.85 (4.73, 10.97) ***P* < 0.001**	10.89 (5.55, 16.23) ***P* < 0.001**	8.88 (4.79, 12.97) ***P* < 0.001**

FAZ, foveal avascular zone.

Data are expressed as the estimates (95% CI).

* Model 1: Adjusted for age and sex.

† Model 2: Further adjusted for axial length, intraocular pressure, mean deviation, retinal nerve fiber layer thickness and image quality score. Bold indicates statistical significance.

Table 4 shows the results of the univariate and multivariate analyses of the factors associated with absolute decreases in CVD. In the univariate analysis, a better image quality score (*P* = 0.009), a faster rate of RNFL decline (*P* < 0.001), and the FI type (all *P* < 0001) were associated with a more rapid rate of CVD decline. In multivariate analysis, faster RNFL decline was independently associated with faster CVD decline (*P* = 0.001) and the FI type (all *P* < 0.001). The factors associated with the CVD percentage rate are shown in Supplementary Table S5.

**Table 4.** Univariate and Multivariate Regression Analysis for Absolute Rate of CVD During Follow-up in Glaucoma

	Univariate Regression		Multivariate Regression	
Characteristics	Coefficient (95% CI)	*P* Value^*^	Coefficient (95% CI)	*P* Value^*^
Age, per year	0.02 (−0.02, 0.06)	0.39		
Systolic blood pressure, per mm Hg	−0.03 (−0.13, 0.07)	0.52		
Diastolic blood pressure, per mm Hg	0.001 (−0.13, 0.12)	0.95		
Intraocular pressure, per mm Hg	0.03 (−0.12, 0.18)	0.66		
Mean deviation, per dB	**−**0.04 (−0.15, 0.08)	0.50		
Pattern standard deviation, per dB	0.01 (−0.21, 0.23)	0.96		
Central corneal thickness, per µm	0.04 (−0.02, 0.11)	0.18		
Anterior central chamber, per mm	−0.18 (−1.67, 1.31)	0.81		
Lens thickness, per mm	−1.70 (−5.33, 1.92)	0.34		
Axial length, per mm	−0.03 (−0.42, 0.36)	0.89		
Image quality score	−0.12 (−0.21, −0.03)	0.009		
Average pRNFL at baseline, per µm	−0.01 (−0.04, 0.03)	0.63		
Average mCT at baseline, per µm	−0.01 (−0.02, 0.001)	0.057		
Changes of pRNFL during follow-up, per µm/y decrease	−0.07 (−0.10, −0.03)	<0.001	−0.05 (−0.09, −0.02)	**0.001**
Changes of mCT during follow-up, per µm/y decrease	0.001 (−0.01, 0.02)	0.92		
Optic disc phenotype				
Focal ischemic	Reference		Reference	
Myopic glaucomatous	4.67 (3.27, 6.07)	**<0.001**	4.47 (3.12, 5.83)	**<0.001**
Senile sclerotic disc	6.51 (4.67, 8.34)	**<0.001**	6.39 (4.51, 8.27)	**<0.001**
Generalized cup enlargement	5.05 (3.22, 6.89)	**<0.001**	3.84 (2.03, 5.65)	**<0.001**

pRNFL, peripapillary retinal nerve fiber layer thickness; mCT, macular choroidal thickness.

* Bold indicates statistical significance.

## Discussion

With a median follow-up of approximately 2.5 years in patients with POAG, we found that CVD decreased more rapidly in the FI type than in the other three phenotypes, in both absolute and percentage rates after correction for confounding factors. A more markedly decreased CVD was associated with more pronounced RNFL thinning.

Recent advances in SS-OCTA have enabled us to make more accurate assessments of choriocapillaris. This study found that the choriocapillaris of FI type declined most rapidly among the four subtypes. It fits with observations made by Nicolela et al. that visual field loss progression and optic disc deterioration were faster in the FI type.^3^ The choroid supplies blood to the entire anterior optic nerve head, and there has been evidence of choroidal vascular damage involved in the progression of the glaucomatous visual field. For example, a fundus fluorescein angiography/ICGA-based study by Laatikainen et al.^29^ found delayed or inadequate filling of the peripapillary choroid in 60% of glaucomatous eyes.^30^ However, it has also been found that there is no significant difference in the rate of peripapillary choroidal thickness thinning between normal and glaucomatous eyes.^31^ There may be some reasons for this controversial result, such as the lack of further phenotyping. Besides, choroidal thickness is not a good indicator of choriocapillaris.^32^ Our study suggests that choriocapillaris plays a role in glaucomatous optic nerve damage in different phenotypes, and it is speculated that the FI type may have a more rapid progression of optic nerve damage because it has a more severe degree of choriocapillaris ischemia. Compared with the other three groups, the initial IOP of type FI was the lowest, whereas the CVD decreased the fastest, which may be suggestive of the optic nerve's susceptibility to non-IOP components. The mechanism may be the loss of photoreceptors due to a reduction in choriocapillaris.^33^^–^^37^

Another finding of our study is that a CVD decrease was independently associated with a decrease in RNFL thickness. The results of previous studies on the relationship between choroidal blood perfusion and RNFL have been controversial. Ho et al.,^38^ Huang et al.,^39^ and Maul et al.^40^ did not find a significant correlation between choroidal thickness and RNFL. However, Gupta et al.^41^ found that thinner peripapillary choroidal thickness was independently correlated with thinner RNFL thickness in the whole circle and the inferior and superior areas. The reason for the controversy may be because these studies were cross-sectional and the choroidal layers include CC, Haller, and Sattler, which implies that the pathophysiological mechanisms are not the same.^32^ The significant correlation between CVD reduction and RNFL thinning rate suggests that more attention should be paid to patients with significantly reduced CVD in a large-scale population screening, especially in the neurodegenerative aspect. Early and more powerful neuroprotective and IOP-lowering treatments should be provided. In addition, CVD parameters can be considered for inclusion in the prediction model to improve the diagnostic and predictive value of GON. However, the specific molecular mechanisms and downstream pathways by which the choriocapillaris plays a role in glaucoma development are currently unknown. This study provides a potential direction for basic research. Animal, molecular, and cellular experiments are needed to elucidate the molecular mechanisms of impaired choriocapillaris, which increases the risk of neurodegenerative vulnerability in patients.

Changes in SVD and DVD did not differ in all the four phenotypes. Presently, only one study has reported differences in retinal blood flow among phenotypes. Ekici et al.^4^ recently analyzed differences in circumpapillary capillary density in 141 glaucoma patients using SD-OCTA, and the SS type had lower peripapillary capillary density compared with the other three types (5% lower than FI and MY, 8% lower than GE). However, this is a study of optic disc blood flow rather than at the macular region, which has been shown to differ between macular and optic discs in glaucoma.^18^ Additionally, this is a cross-sectional study, and there are no longitudinal vascular changes of the four phenotypes. This study first found that the difference of SCP and DCP among the four phenotypes in longitudinal changes was not significant, with the only CVD being significantly different among the four phenotypes. This suggests that choriocapillaris is more imperative in glaucomatous optic nerve damage than SVD and DVD. Thus CVD is thought to be a more sensitive early biomarker for detecting glaucoma progression. Alternatively, ethnic differences may explain the difference in results. It has been shown that there are significant ethnic differences in choroidal thickness and blood flow measured by OCTA in different ethnic groups.^42^^–^^45^ Overall, whether choroidal ischemia causes optic nerve damage or whether less demand for blood supply because of optic nerve damage induces this association between them needs to be investigated in future studies.

The proportion of female participants in this study was relatively low. Previous studies have reported a significantly higher prevalence of POAG in males than in females.^46^^–^^48^ It has been speculated that sex plays a role in the incidence of POAG, which may be related to the effects of sex hormones on IOP, ocular blood flow, and neuroprotection. Estrogen is associated with increased ocular blood flow, decreased IOP, and neuroprotection.^49^ Thus estrogen-induced vasodilation and its effect on the outflow of aqueous humor may contribute to the low prevalence of POAG in women, which was also indirectly confirmed in our study.

Our study has some limitations. First, the small sample size may have compromised statistical power. From a statistical perspective, there were positive results in this study, which means that increasing the sample size does not increase the type I error. Increasing the sample size will only have an impact on negative results (*P* > 0.05) and will not change positive results. Clinically, we found that FI patients with well-controlled IOP had the fastest progression of visual field compared to the other three types of glaucoma, which is consistent with previous reports.^3^ This could be evidence suggestive of the susceptibility of the optic nerve to non-IOP components. Regardless of this, studies with larger sample sizes should be conducted to illustrate the change rates in the choriocapillaris at the patient level for different phenotypes. Second, given the small sample size of the SS and GE groups, the choriocapillaris change rates might be unrepresentative. Future studies with larger sample sizes in the SS and GE groups are needed to validate these findings. Third, the follow-up period was relatively short, with a median follow-up time of 2.5 years. A longer follow-up period for the four groups of patients would have allowed a better assessment of the relationship between glaucoma and CVD over time. However, most clinical decisions were made in a relatively short time, usually within 1.6 years.^31^^,^^50^^,^^51^ Fourth, because we included only all POAG, this finding cannot be applied to other types of glaucoma, such as angle closure glaucoma and other types of open-angle glaucoma, including pseudoexfoliative glaucoma. Fifth, the low number of females with POAG included in this study may be related to sex differences in POAG prevalence. Nevertheless, a more gender-balanced cohort study is needed in the future. Sixth, although the IOP values of the final enrolled patients remained stable within the range of 10 to 21 mm Hg throughout the follow-up period, we could not confirm whether the nocturnal IOP increased in these patients, because previous studies have found significant variations in diurnal IOP in patients with glaucoma.^52^^,^^53^ Further evaluation of 24-hour IOP is needed in the future to better facilitate the assessment, management, and treatment of glaucoma patients.

Strengths of the study are that first, the use of SS-OCTA for different phenotypes of glaucoma allowed for better visualization of the CVD. Second, this study has used the choriocapillaris imaging guideline^23^ for measuring CVD. Third, it is a prospective longitudinal study design with a follow-up period of at least one and a half years. Fourth, this study only included Chinese patients with POAG, which implies good homogeneity of the population. To our knowledge, this is the first longitudinal study to analyze the changes and associations of choriocapillaris perfusion in different optic disc phenotypes using SS-OCTA.

In conclusion, four glaucomatous optic disc phenotypes showed differences in the CVD decrease, with the decrease being most marked in the FI type. The CVD decrease correlates independently with the rate of RNFL thinning. Future longitudinal studies that include different ethnic populations, larger sample sizes, and longer follow-up times are needed to validate these findings further.

## Supplementary Material

Supplement 1
