# Supplementary material for: Longitudinal Choriocapillaris Vascular Density Changes in Different Types of Primary Open-Angle Glaucoma
Source: Transl Vis Sci Technol. 2023 Jan 18;12(1):21. doi: 10.1167/tvst.12.1.21 (PMC9855283; doi:10.1167/tvst.12.1.21)
Supplement: Supplement 1 [file tvst-12-1-21_s001.pdf]

Supplemental Table 1. Average number of images included in the final analysis in each group during the follow-up period.

	All	FI	GE	MY	SS	p-value
Mean (SD)	7.16(1.13)	6.97(1.15)	7.38(1.04)	7.62(0.87)	6.62(1.33)	0.078
25%	6	6	7	7	6	-
75%	8	8	8	8	7	-

p-value is calculated by the method of one-way ANOVA. SD= Standard Deviation. FI= focal ischemic type; MY= myopic type; SS= senile sclerotic type; GE= generalized enlargement type.

Supplemental Table 2. Measurements of baseline of retinal and choroidal vascular parameters in four glaucomatous optic disc phenotypes.

	**FI**	**MY**	**SS**	**GE**	**P-value***
FAZ, mm^2	0.30(0.10)	0.40(0.14)	0.31(0.10)	0.37(0.10)	**0.013**
SVD, %	41.64(1.77)	40.84(1.68)	40.35(1.58)	41.07(1.77)	0.089
DVD, %	38.52(2.30)	38.98(2.41)	36.86(3.02)	37.85(2.81)	0.059
CVD, %	60.82(2.11)	59.82(1.72)	57.79(4.03)	59.56(2.71)	**0.003**

Data are expressed as the mean (SD). FI= focal ischemic type; MY= myopic type; SS= senile sclerotic type; GE= generalized enlargement type; FAZ= foveal avascular zone; SVD= superficial vessel density; DVD= deep vessel density; CVD= choriocapillaris vessel density. *Bold indicates statistical significance.

Supplemental Table 3. The number of OCTA visits and follow-up duration in different optic disc phenotypes.

	All	FI	GE	MY	SS	p-value
Follow-up, m	26.31(10.06)	26.31(9.73)	26.47(9.91)	26.74(10.28)	26.04(10.80)	0.993
Number of visits	8.77(3.35)	8.76(3.24)	8.82(3.30)	8.91(3.43)	8.68(3.60)	0.992

p value is calculated by the method of one-way ANOVA. SD= standard deviation. FI= focal ischemic type; MY= myopic type; SS= senile sclerotic type; GE= generalized enlargement type.

Supplemental Table 4. Changes of choriocapillaris vessel density in four glaucomatous optic disc phenotypes after adjusted for potential confounder.

	MY vs SS	GE vs SS	MY vs GE
**Model 1 ***			
Absolute rate	-2.42(-4.32,-0.53)	-1.82(-3.99,0.36)	-0.61(-2.41,1.19)
	**p=0.013**	p=0.101	p=0.504
Percent rate	-4.50(-7.87,-1.13)	-3.39(-7.26,0.48)	-1.11(-4.32,2.1)
	**p=0.009**	p=0.086	p=0.493
**Model 2 †**			
Absolute rate	-1.78(-4.99,1.43)	-1.31(-4.73,2.12)	-0.47(-3.07,2.12)
	p=0.270	p=0.445	p=0.715
Percent rate	-3.04(-8.42,2.33)	-2.01(-7.74,3.73)	-1.03(-5.38,3.31)
	p=0.260	p=0.484	p=0.634

*Model 1: Adjusted for age and sex.

†Model 2: Further adjusted for axial length, intraocular pressure, mean deviation, retinal nerve fiber layer thickness and image quality score. Bold indicates statistical significance.

Supplemental Table 5. Univariate and multivariate regression analysis for percentage rate of choriocapillaris vessel density (CVD) during follow-up in glaucoma.

**Characteristics**	**Univariate regression**		**Multivariate regression**	
	**Coefficient (95% CI)**	**P-value***	**Coefficient (95% CI)**	**P-value***
Age, per year	0.03(-0.04,0.10)	0.413		
Systolic blood pressure, per mm-Hg	-0.05(-0.23,0.13)	0.591		
Diastolic blood pressure, per mm-Hg	-0.02(-0.25,0.21)	0.878		
Intraocular pressure, per mm-Hg	0.05(-0.20,0.30)	0.696		
Mean deviation, per dB	-0.07(-0.26,0.13)	0.492		
Pattern standard deviation, per dB	0.01(-0.36,0.39)	0.940		
Central corneal thickness, per µm	0.08(-0.04,0.19)	0.170		
Anterior central chamber, per mm	-0.36(-2.93,2.21)	0.781		
Lens thickness, per mm	-3.16(-9.49,3.17)	0.313		
Axial length, per mm	-0.04(-0.72,0.64)	0.907		
Image quality score	-0.22(-0.37,-0.07)	0.004		
Average pRNFL at baseline, per µm	-0.02(-0.08,0.04)	0.573		
Average mCT at baseline, per µm	-0.01(-0.03,0.001)	0.088		
Changes of pRNFL during follow-up, per µm/y decrease	-0.12(-0.18,-0.06)	**<0.001**	-0.10(-0.16,-0.04)	**0.001**
Changes of mCT during follow-up, per µm/y decrease	0.002(-0.03,0.02)	0.817		
**Optic disc phenotype**				
Focal ischemic	Reference		Reference	
Myopic glaucomatous	7.74(5.26,10.22)	**<0.001**	7.40(5.01,9.80)	**<0.001**
Senile sclerotic disc	11.22(7.96,14.47)	**<0.001**	11.1(7.78,14.43)	**<0.001**
Generalized cup enlargement	8.46(5.21,11.72)	**<0.001**	6.28(3.08,9.49)	**<0.001**

pRNFL= peripapillary retinal nerve fiber layer thickness; mCT=macular choroidal thickness.

*Bold indicates statistical significance.

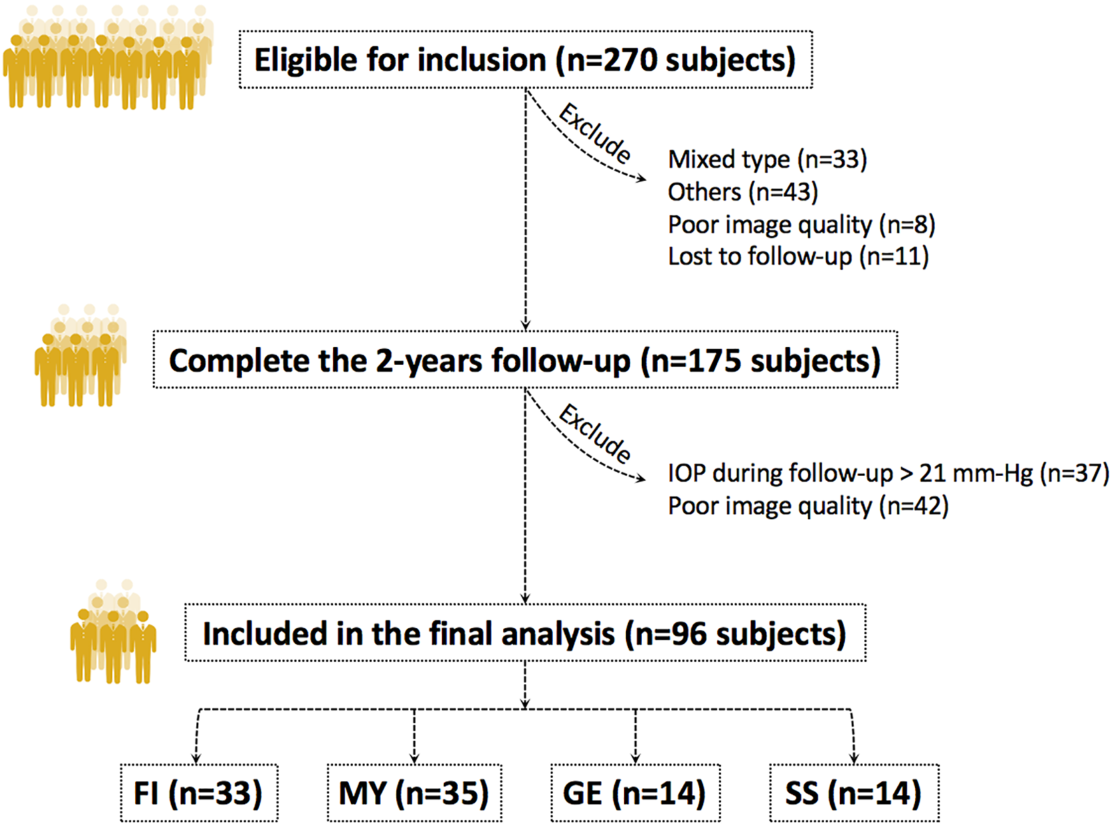

Supplemental Figure 1. Flow chart of the longitudinal study design (n= 96).
